# A Critical Assessment of the Congruency between Environmental DNA and Palaeoecology for the Biodiversity Monitoring and Palaeoenvironmental Reconstruction

**DOI:** 10.3390/ijerph19159445

**Published:** 2022-08-01

**Authors:** Shahnawaz Hassan, Zulaykha Khurshid, Sabreena, Bikram Singh Bali, Bashir Ah Ganai, R. Z. Sayyed, Peter Poczai, Muzafar Zaman

**Affiliations:** 1 Department of Environmental Science, University of Kashmir, Srinagar 190006, India; shahnawazhasssan89@gmail.com (S.H.); zulaykhazehra@gmail.com (Z.K.); bhatsabreen32@gmail.com (S.); muzafarzaman@gmail.com (M.Z.); 2 Department of Earth Sciences, University of Kashmir, Srinagar 190006, India; baligeol@gmail.com; 3 Centre of Research for Development, University of Kashmir, Srinagar 190006, India; 4 Department of Microbiology, PSGVP Mandal, s, SI Patil Arts, G B Patel Science and STKV Sangh Commerce College, Shahada 425409, India; sayyedrz@gmail.com; 5 Finnish Museum of Natural History, University of Helsinki, 00100 Helsinki, Finland

**Keywords:** environmental DNA, biodiversity monitoring, sampling design, biodiversity conservation, palaeoenvironmental reconstruction

## Abstract

The present study suggests that standardized methodology, careful site selection, and stratigraphy are essential for investigating ancient ecosystems in order to evaluate biodiversity and DNA-based time series. Based on specific keywords, this investigation reviewed 146 publications using the SCOPUS, Web of Science (WoS), PUBMED, and Google Scholar databases. Results indicate that environmental deoxyribose nucleic acid (eDNA) can be pivotal for assessing and conserving ecosystems. Our review revealed that in the last 12 years (January 2008–July 2021), 63% of the studies based on eDNA have been reported from aquatic ecosystems, 25% from marine habitats, and 12% from terrestrial environments. Out of studies conducted in aquatic systems using the environmental DNA (eDNA) technique, 63% of the investigations have been reported from freshwater ecosystems, with an utmost focus on fish diversity (40%). Further analysis of the literature reveals that during the same period, 24% of the investigations using the environmental DNA technique were carried out on invertebrates, 8% on mammals, 7% on plants, 6% on reptiles, and 5% on birds. The results obtained clearly indicate that the environmental DNA technique has a clear-cut edge over other biodiversity monitoring methods. Furthermore, we also found that eDNA, in conjunction with different dating techniques, can provide better insight into deciphering eco-evolutionary feedback. Therefore, an attempt has been made to offer extensive information on the application of dating methods for different taxa present in diverse ecosystems. Last, we provide suggestions and elucidations on how to overcome the caveats and delineate some of the research avenues that will likely shape this field in the near future. This paper aims to identify the gaps in environmental DNA (eDNA) investigations to help researchers, ecologists, and decision-makers to develop a holistic understanding of environmental DNA (eDNA) and its utility as a palaeoenvironmental contrivance.

## 1. Introduction

The greatest challenge in the 21st century is the incessant diminution of Earth’s biodiversity, as the populations of world biota are being depleted at a greater rate than in pre-human periods [1,2,3,4,5,6]. Despite the fact that information on biodiversity is limited or even non-existent for many species and geographical locations, there is a worldwide political consensus to prevent the present biodiversity loss [7,8]. Smaller disintegrated populations are at increased risk of extinction due to habitat destruction by enhanced demographic, genetic, and environmental haphazardness. Though, such smaller natural populations vulnerable to extinction, as established by experimental investigations, are finite [9]. However, biological assessment and monitoring to acquire accurate species data apportionment on an ecological and political timescale becomes an essential conservation exertion to save rapidly diminishing biodiversity [10].

Visual surveys and morphological characterization of species have been the conventional method of choice for biological surveillance over a period of time. Although these methods are highly reliable when species are evident and abundant, reliability is compromised when organisms are present in low densities, thereby inflating error and biased parameter estimates [11,12]. Due to morphological plasticity and closely related species with extremely similar appearances in the juvenile stages, these methodologies may fall short of executing efficient and uniform surveys in certain circumstances. As a result, there are instances of species databases including inaccuracies. Furthermore, conventional monitoring approaches, such as marine surveys that rely on extremely destructive procedures, have occasionally proved to be invasive on the species or environment under investigation [12]. In many cases, what has been noticed is a flawed species database with errors in morphological identification, considerably highlighting the importance of taxonomic expertise, which is often not of the highest quality or is in expeditious decay currently. Therefore, there is a need for alternative and innovative methods to overcome the limitations of traditional biodiversity surveillance techniques.

Critical development for accelerated research efforts is periodically stimulated by scientific advancements considerably altering scientific thought. One such phenomenon of cardinal prominence is the discovery of eDNA, i.e., genetic material obtained directly from environmental samples (soil, sediment, water, etc.) to inscribe ecological questions [13]. Environmental DNA, a prime component of the ecologist’s and environmental manager’s toolbox, is composite genetic DNA originating from the environmental specimen with no self-explanatory signs of biological acquired material [14,15] for the discovery of the most all-encompassing DNA-based taxonomic or functional information for the ecosystem under consideration [16]. Environmental DNA has been a novel technique for biodiversity monitoring, palaeoenvironmental reconstruction [17], and spatio-temporal shifts in ecosystem diversity [18]. Moreover, eDNA has widespread utility in species biomonitoring in a wide variety of habitats such as desert springs [19], lagoons [20], arctic [21], seawater [22], forests [23], ponds [24], estuarine sediments [25], and permafrost [26]. Studies have also reported that eDNA surveys provide complementary information, particularly for the management of important inland fisheries [27]. Investigations have also revealed that eDNA metabarcoding detects more species than traditional survey methods such as gillnet surveys [28]. Similarly, it is a fast and broad biodiversity survey method that can dispense an exhaustive review of species in extremely heterogeneous tropical marine coral reefs [29]. The scope of environmental DNA is limited by DNA degradation, affecting the inference of fine-scale species and community’s spatio-temporal trends. However, despite these shortcomings, environmental DNA has tremendous potential in conservation, biodiversity monitoring, and ecosystem assessment, provided the procedure is optimized, standardized, and unified, thereby integrating taxonomy and molecular methods for any ecological study.

Even if methodological improvements are still needed [14,15], an environmental sample may be acquired in a highly consistent way across locales in a particular kind of habitat. This is more challenging using conventional approaches since outcomes are often dependent on the taxonomic expertise and experience of survey employees. eDNA is a completely non-invasive technology that does not harm the animals or environments being studied. Even high-quality taxonomic competence is sometimes insufficient in cases where the species of conservation interest have cryptic life patterns or requires the study of juvenile life stages that are difficult to distinguish from closely related species. In this case, eDNA approaches may outperform conventional methods in detecting species. It would be incorrect to generalize that eDNA is less expensive than conventional approaches since this depends on the target species. Nonetheless, multiple studies show that utilizing eDNA saves time and money when compared to conventional monitoring procedures [11,12]. As the cost of sequencing each base pair continues to fall exponentially, eDNA will likely outperform some existing approaches, particularly when adopting a metabarcoding approach. Traditional surveys are problematic for numerous species (e.g., amphibians) outside of certain seasons or weather circumstances when adult vocal activity is at its highest. eDNA, on the other hand, may persist in their ecosystem outside of these high-activity times (e.g., from juveniles), increasing the duration for monitoring. Harsh weather conditions may limit the use of conventional fishing equipment for other species (such as fish) but not eDNA sampling. The advancement of DNA sequencing technology has substantially extended the applications of eDNA and is predicted to continue further improvements in the future. Finally, we seek the most complete method of using eDNA for the benefit of our planet and all of its people. Environmental DNA will essentially be a tool for monitoring biodiversity, providing quick and efficient insights into species distribution, abundance estimates, and, eventually, population numbers, all of which will serve as the foundation for proper conservation activities. As a result, it will never directly address the biodiversity catastrophe, which is a more challenging issue that requires, particularly, political will, commitment, and action.

The concept of eDNA not only finds its applicability in monitoring the health and eminence of modern ecosystems but also provides a fascinating way to reconstruct past environments to provide a complete overview of ancient ecosystems. For future conservation planning, such reconstructed ecosystems can be utilized for backward testing of climate change models, emergence tracking of invasive species, and the valuation of anthropogenic impacts on ancient biodiversity and landscape [30].

In this review, we highlight the primary outcomes of the eDNA technique for specifying high-resolution community alignment for biodiversity surveillance and its nexus with ancient environment reconstruction via dating techniques that can be used with environmental DNA to explicate more knowledge for better management and conservation of global biodiversity. This review attempts to develop a better understanding of the concept of eDNA as a novel tool for biodiversity monitoring, its limitations, and possible solutions to achieve desirable and accurate results.

## 2. Methodology

### 2.1. Literature Search

To highlight the prominence and application of environmental DNA, an extensive literature review was undertaken using diverse scientific databases (PubMed, Web of Science, Scopus, and Google Scholar) by adopting a keyword-based search (Table 1). Keywords used include eDNA, environmental DNA, palaeoenvironmental reconstructions, biodiversity monitoring, and conservation. The article search was limited to the past 12 years (January 2008–July 2021) to extract and explore the recent progressions and findings in eDNA research. These searches were filtered using a thorough study of their abstracts, results, and conclusions to achieve the objective of the review.

### 2.2. PRISMA Analysis

**Figure 1:**
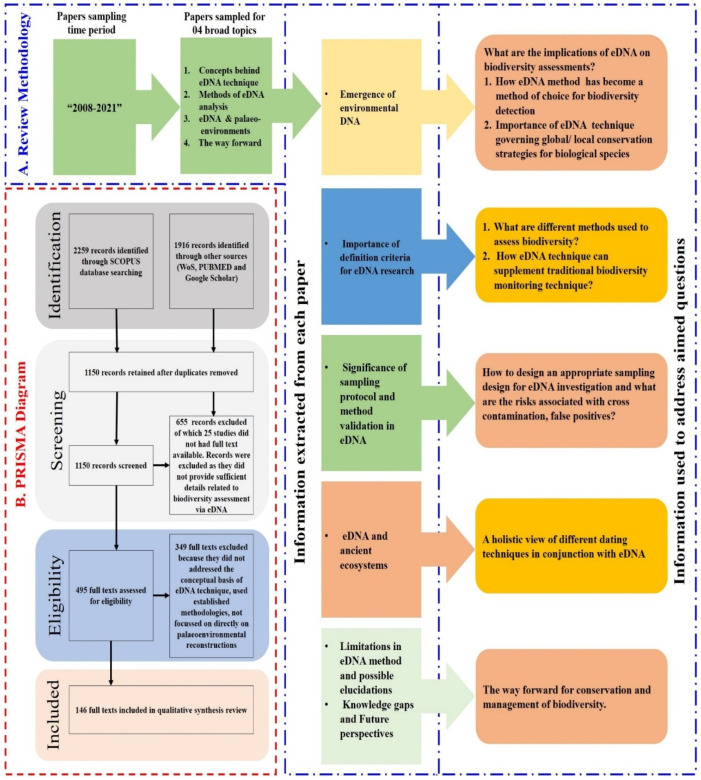
Overview of the review methodology. (**A**) Literature review design, information collected, and research questions. (**B**) PRISMA methodology flow chart showing the selection and rejection of papers. Of the original 4175 articles, 495 were evaluated for eligibility based on the relevant questions, aims, and objectives.

We followed the PRISMA protocol for this systemic review, as shown in Figure 1B. Furthermore, we mostly focused on themes such as the emergence of environmental DNA, sampling design optimization, eDNA, palaeoenvironmental reconstructions, problems associated with environmental DNA techniques, and possible elucidations. After rigorous literature searches and tediously following the protocol, we finally shortlisted 146 publications that fulfilled the above-mentioned aim and themes we framed for this review.

## 3. Sampling Design Optimization and Method Validation

For any ecological investigation, an appropriate sampling design is crucial. Due to the dearth of a universally trusted perspective, the sampling strategy should be adjusted as per the type of environment, the scientific question, and financial, logistical, and statistical considerations [15]. Pilot study results eventually become very important from the sampling point of view. For eDNA analyses, the exact procedure should be followed. In case of any ambiguity regarding experimental problems arising from eDNA analysis, it is better to carry out pilot experiments, which can help in structuring a reliable experiment to vanquish the errors that may arise during sampling, eDNA extraction, PCR sequencing, and data interpretation. This setup will provide errorless results [15].

The standardization of the procedure for minimum sample degradation is another facet to bear in mind while designing the sampling strategy for eDNA studies. Biological replicates are crucial for every study so that the estimation of variability introduced during sampling can be interpreted correctly [31]. Prior knowledge about the origin, fate, and transport of eDNA is also an essential consideration to be kept in mind while structuring the sampling strategy [32]. One should decide whether the sampling should be stratified or pooled as it can give variable results even in the same habitat. So, to fully ascertain the complete diversity in heterogeneous environments, samples should be gathered in different sections. One should also decide whether sieving or filtering of the sample is needed or not [33,34]. In sifting soil or sediment soil, for example, there will be a greater proportion of microorganisms and propagules (pupas, pollens, or seeds), producing a bulk sample enriched in meiofauna and root tissues providing extortionate quality DNA as most inorganic and humic substances are excluded as reported for benthic meiofauna [35] or plants [36]. An imperative aspect to consider during sampling is the appropriate magnitude of the investigation area and its resolution for capturing the environmental heterogeneity of the area under consideration. It will help in describing patterns, estimating diversity parameters, or detecting species.

### 3.1. Delineating the Sample Strategy

There are different sampling designs that can be employed in different environmental settings. Systematic, random, and stratified sampling designs can be employed on soil samples. When the studied area displays linear environmental gradients, the appropriate approach that can be used is systematic sampling [37,38]. If the objective of the investigation is to analyze the distance decay (as the distance between samples increases, the level of similarity decreases), a systematic model with a logarithmic spacing of representative units is most appropriate. Another benefit of the systematic strategy is that it can be employed on transect (linear or belt). Ramirez et al. (2014) utilized the technique of random sampling for ecological investigation using the eDNA technique. Stratified sampling is applied to heterogeneous environments entailing discrete entities, allowing improved sample depiction by reducing the sampling error.

Contemporary scientific inquiries have also defined the sampling strategy based on climatic or biological space modeling, which has proven more proficient in delineating the biological patterns of interest [39,40]. Sampling stratum with an equal quantity of samples in habitats with high contrasted diversity and heterogeneity might not be appropriate, so in such cases, the sampling representativeness in each habitat has to be maximized [41]. It is critical that the same effort and related inferential pitfalls must apply to eDNA as they do to traditional species survey methods [42]. In case of collection of water per sample, the best recommendation is to collect water from the proposed sample site and determine how much can be pushed through filters of various pore sizes or how much water can be centrifuged. It has been reported that a 2 L water sample with glass microfiber with a pore size of 1.5 μm worked best in the search for Asian carp [43]. Similarly, 500 mL samples with a 0.22-μm filter capsule worked for the detection of white sharks in southern California [44].

### 3.2. Sediment Coring

Similarly, while dealing with deep sediments, sediment cores serve as an excellent setup for demonstrating the sedimentation rate, the history of pollutant additives to the water systems, and as inventories of contaminants that could prove vital to the comprehension of species dynamics and surveillance. In the last decades, extensive demand for sediment coring has ensued rapid progression of sampling techniques. Studies have revealed that the sediment core is subjected to various disturbances and may not always elucidate the in situ sediment characteristics [45]. Different corers utilized for sample extraction can eventually be employed for contaminant history inputs to preserve the sediment structural integrity and ambient (in situ) conditions. The extent of exogenous contamination penetration in the palaeoenvironmental retrieved core can be determined using genetic tracers when coring, as indicated by many palaeoenvironmental investigations [26,46,47]. Genetic tracers have been widely utilized for monitoring contamination during the coring process by introducing the laboratory strains of *Serratia marcescens* bacteria around the drilling apparatus [48]. Similarly, scientific investigations have employed perfluorocarbon tracers during the drilling process to test the cores on board for coring and sampling contamination [49]. Fluorescent plastic microspheres and *Pseudomonas putida* genetically tagged with green fluorescent protein production have been used to mimic potential microbial contamination of permafrost cores [50].

Incision of at least 1 m of the sampling surface should ideally be carried out to lessen the possibility of recent DNA deposition [51]. Intra-site and inter-site variation can be examined by employing parallel sampling, allowing multiple-sample analysis from the same deposit, ensuring palaeoecological regenerations are not distorted [52]. While investigating sediment cores, an outside portion (1–3 cm) should be removed owing to its exposure to exogenous contaminates during the coring process [51]. Sub-samples from the center, base, and top should be selected to elude contaminations on older layers by the younger DNA.

### 3.3. Inclusion of Appropriate Controls and Sample Preservation

To detect very low levels of DNA, most molecular ecologists include negative controls in PCR reactions. Negative controls typically account only for contamination in the laboratory. However, accounting for contamination in the field may be more important and challenging. In order to gain insight into the potential contamination, field equipment should be sampled through swabbing. Similarly, sample media and containers can also be tested. The use of negative controls in the laboratory stages of analysis is crucial to maintain sample integrity. During PCR, the low affinity between the primer and primer-binding sites results in false negative detections. To minimize PCR biases, thorough in silico and in vivo evaluation of universal primers is needed to test their biodiversity coverage before their use. Therefore, the DNA metabarcoding protocol should be fitted to geographical regions and taxonomic interest groups. After the sample collection, DNA degradation occurs or the microbial community changes over time, so it is better to extract DNA as quickly as possible after sampling. On the other hand, it is quite difficult to achieve this in the field, so blocking all biological activities as quickly as possible becomes mandatory in eDNA investigations. This can be achieved by freezing the sample in liquid nitrogen or a freezer. However, it might impact the DNA extraction process by targeting extracellular DNA, as cell lysis can occur during the storage process. On the other hand, if total DNA is the target, then the freezing/unfreezing process can facilitate cell lysis and subsequent DNA extraction.

## 4. Results and Discussion

### 4.1. Conceptual Background and Emergence of eDNA

**Figure 2:**
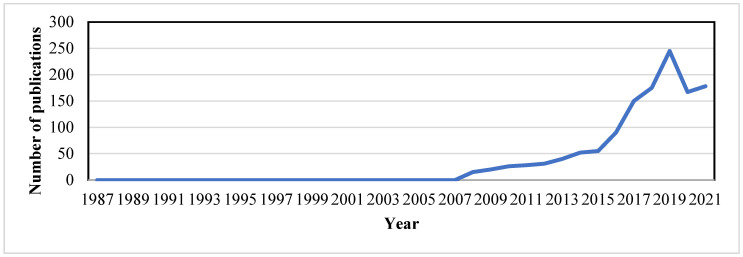

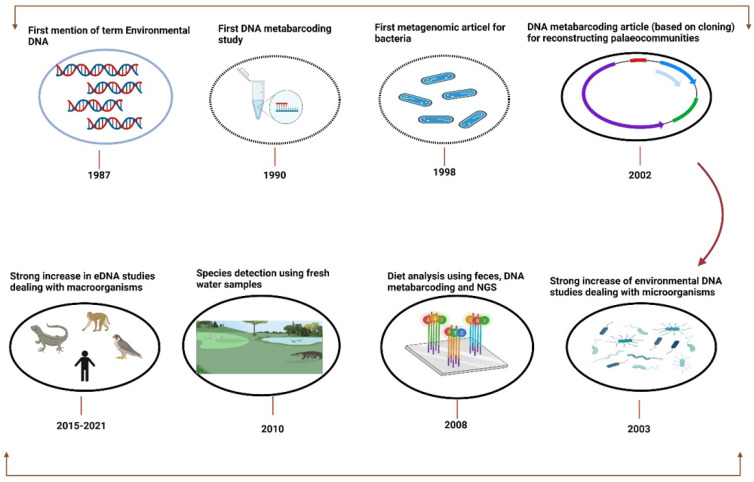
Overview and emergence of environmental DNA as a tool for biodiversity monitoring from “2008–2021”.

The first report on the eDNA extraction protocol was published in 1987 by Ogram et al. (1987), where lake sediments were used to investigate microbial DNA (Figure 2). Within a short span of 3 years after the first DNA metabarcoding research investigation was disseminated and analyzed, the diversity of the 16S rRNA gene in bacterioplankton sampled from the Sargasso Sea using PCR amid cloning was published in the year 1990 [53]. For the synthesis of bioactive molecules in uncultivated microorganisms, the metagenomics technique was used by Handelsman et al. (1998), where cloning and sequencing of soil eDNA fragments were carried out to discern new pathways. A noteworthy study on DNA metabarcoding was published in 2003, which elucidated the retrieval of megafaunal (mammoth, Bison horse) and ancient plant DNA from permafrost and DNA of extinct ratite moa from cave sediments [48]. The expensive and time-consuming cloning step was made superfluous by the tumult of next-generation sequencing (NGS) after 2005 [54]. By 2010, DNA barcoding was stretched out to macroorganisms for diet analysis [55,56] and then for water and soil eDNA studies [57,58]). Recently there have been numerous publications on freshwater microorganisms concerning single species detection [24,59,60,61,62]. Over the years, different methods have been applied for eDNA analysis, for targeting single species, and standard or quantitative PCR for detecting all taxa from a given taxonomic group. PCR-based assays are vital, such as for bacteria [34,63], fungi [64,65], plants [58,66,67], eukaryotes [25,68,69], fish [62,70,71,72,73], etc.

**Figure 3:**
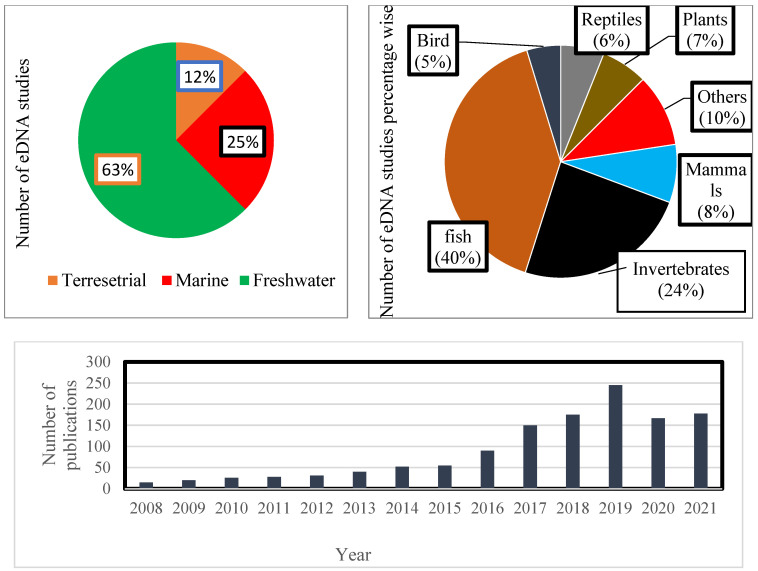
Using keywords ‘environmental DNA’, ‘eDNA’, ‘environmental DNA’, and ‘palaeoenvironmental reconstruction’: the number of studies that contained one of these terms in their title, the keywords, or the abstract recovered from a literature search during the period between 1 January 2008 to July 2021.

In contemporary times, while analyzing the literature, it has been found that environmental DNA is a tool of wide application for studying freshwater ecosystems (63%) followed by marine (25%) and terrestrial habitats (12%). From these habitats, fish diversity has been widely studied, followed by invertebrates and mammals (Figure 3). Studies aiming to distinguish multiple taxa through metagenomics and metabarcoding have also been reported [22,72] and have proved to be an attractive approach, gaining insight into the past communities typically after 2014 [74,75,76]. Notwithstanding a very innovative approach at the bench and bioinformatics level, some other technical aspects remain highly challenging.

### 4.2. Species Biomonitoring through Environmental DNA

Spear et al. (2021) conducted a study on the freshwater ecosystem for biodiversity detection and revealed that the environmental DNA technique can dispense knowledge that might be key for the management and conservation of lakes. The correlation observed in this study is considered second to none, as, in this study, it was demonstrated that eDNA and population abundance has a strong relationship compared to traditional species surveys [27]. Comparison of the detection sensitivity with traditional surveillance methods is fundamental to making eDNA study a concrete biodiversity monitoring tool. Afzali et al. (2021) applied the concept of environmental DNA to an estuarine ecosystem in Canada. This study utilized a traditional probing assessment of the demersal fish community to compare the result with eDNA metabarcoding. This study revealed about 53% concurrences in the estimation of species detection concerning eDNA metabarcoding in combination with trawl surveys. The combination of these methods showed a significant correlation in estimating relative abundance; however, the relationship was affected by environmental factors (temperature, depth, salinity, and oxygen) [77]. Overall, it has been established that eDNA can deliver more sensitive surveillance outcomes that are improved in comparison to traditional surveys. A similar type of study was reported from Rupert River, Canada, where it was reported that eDNA metabarcoding detected more fish species [28]. In addition, the environmental DNA technique detects a diverse range of taxonomic groups as compared to traditional surveys, as indicated by the investigation conducted by Polanco Fernadez et al. (2021) in Colombia. This study indicated that eDNA provides inclusive knowledge about the fish diversity in highly mixed tropical coral reefs. A study by Thalinger et al. (2021) revealed the spatio-temporal shifts in riverine ecosystem diversity as seasonal changes affect the eDNA distribution. To make a valid and accurate inference in both time and space for the studied species, it is highly pertinent to understand the behavior of DNA in the environment. This is because the detection process by eDNA varies seasonally in lotic environments, making parameters such as hydrological conditions and traits of species extremely important to make inferences in time. Similarly, in desert springs, the eDNA method provides better insight into species recovery, as indicated by the investigation by Mejia et al. (2021).

Various studies have been conducted in different habitats such as lagoons [20], arctic [21], pelagic diversity in marine ecosystems [78,79,80], stream biodiversity [81], seawater [22], lake sediments [82,83], and permafrost [84]. All these investigations have indicated that eDNA can be a method of choice for species identification. Such a technique has greater reliability and reflects more information and species resolution. Forest and pond ecosystems have also been studied for the mammalian diversity and distribution of invasive species. The investigation carried out by Calvignae et al. (2013) utilized Caryion fly-derived DNA to detect mammalian diversity. Similarly, invasive species presence has also been studied using the eDNA technique by Takahara et al. (2013). Dejean et al. (2012), in their study on the American *bullfrog*, indicated that the environmental DNA technique has a better sensitivity and reduces sampling time and effort. Similarly, Ficetola et al. (2008), while applying the eDNA method on *Rana catesbeiana*, a frog species, also revealed higher species-specific detection from environmental samples. An increasing number of studies have showcased the tremendous potential of eDNA technology for providing new insights into the species ecology, moving beyond species detection occurrence and community description [85]. While going through the literature review on eDNA, it can be stated that it also has notable usefulness as a practical tool for studying fish reproductive biology [86]. Some selected studies (January 2008–July 2021) enlightening the applicability of environmental DNA as a biodiversity monitoring tool are summarized below with their respective sub-titles (Table 2).

The above studies reveal that eDNA has a clear edge over other biodiversity monitoring methods. These studies indicate that environmental DNA analysis can be a powerful non-invasive detection method, posing higher reliability in the diversity of systems and across the branches of the tree of life [87,88,89,90]. Progression of eDNA as a tool to measure broad species diversity and population has also been investigated in various contributions. The applicability of eDNA as a novel tool to evaluate the efficiency of restoration and/or management strategies is emerging at an impressive amplitude to provide faster and potentially reliable monitoring [91,92,93]. The coupled approach of eDNA detection and conventional/traditional survey methods can have several complementary advantages. However, due to the lack of standardization guidelines, those advantages are sometimes undermined. The requisite guidelines, if adopted, will allow greater precision and accuracy in data comparison from multiple monitoring sites at varying points in time.

**Table 2:** A sample of research publications that focused on environmental DNA as a species monitoring technique.

References	Habitat/Ecosystem	Representative Species	Use	Major Findings
Spear et al. (2021) [27]	Freshwater	*Sander vitreus*	Assessing population abundance	eDNA monitoring can appropriately dispense lakes to real world management categories for early warning for at-risk lakes in need of attention.
Afzali et al. (2021) [77]	Estuary	Demersal fish communities	Monitoring species biodiversity	eDNA metabarcoding out-competes traditional survey methods by enabling detection of rare and endangered taxa.
Boivin-Delisle et al. (2021) [28]	Freshwater	*Sander vitreus*	Species-specific biomonitoring	eDNA technique based on species-specific primers can provide insightful cognizance on fish biodiversity.
Polanco Fernández et al. (2021) [29]	Tropical marine coral reefs	*Actinopterygii* and *Elasmobranchii*	Species-specific biomonitoring	eDNA approach can provide an inclusive outline of fish composition in highly assorted coral reefs.
Capo et al. (2021) [17]	Lake sediments	Aquatic community	Biodiversity monitoring and palaeoenvironmental reconstructions	Despite a lack of clear and concise guidelines regarding sediment ancient DNA (SedaDNA), future SedaDNA research will provide more robust and result-oriented information about palaeoenvironments.
Thalinger et al. (2021) [18]	Riverine	*Phoxinus phoxinus* *Salmo trutta* *Oncorhynchus mykiss* *Salvelinus fontinalis*	Spatio-temporal shifts in ecosystem biodiversity	Seasonal discharge conditions prompt deep lateral and longitudinal changes in eDNA distribution.
Tsuji & Shibata (2021) [86]	Freshwater	*Oryzias latipes* *Oryzias sakaizumii*	Reproductive biology	Spawning events spike eDNA concentration, which offers the prospect to monitor and comprehend spawning timings with less effort than traditional methods.
Mejia et al. (2021) [19]	Desert springs	Plant and animal	Species recovery	eDNA is a promising supplemental tool to traditional approaches for biodiversity monitoring in desert springs.
Oka et al. (2021) [20]	Lagoon	*Enneapterygius philippinus* *Spratelloides delicatulus* *Rhabdoblennius nitidus* *Enneapterygius similis*	Biodiversity monitoring	For estimation of species diversity in tropical and subtropical areas, eDNA is a useful, rapid, and cost-effective method.
Székely et al. (2021) [21]	Arctic	*Balaena mysticetus*	Genetic diversity	Cetacean footprints are a promising cradle of genomic DNA.
Agerbo Rasmussen et al. (2021) [80]	Experimental vineyard	Fungi and arthropods	Species biomonitoring	eDNA offers a context for diversity assessment in vineyards to make more universal conclusions.
Shu, Ludwig, & Peng (2020) [73]	Freshwater	Freshwater fish *Misgurnis anguillicaudates* *Cyprinus carpio* *Salvelinus fontinalis*	Quantification	Despite its methodological obstacles, eDNA remains a promising and powerful contrivance for fish monitoring and conservation.
Zhang et al. (2020) [79]	Marine	Bacteria and marine mammals	Pelagic diversity	eDNA-based metabarcoding has the potential for successful multiple biodiversity surveillance, offering technical support and knowledge for future ecosystem protection and resource reservation.
Jeunen et al. (2019) [78]	Marine	Multi-specific	Species-specific biodiversity	The DNA extraction protocols when corrected and optimized provide clear illustration of eDNA monitoring in the marine environment.
Li et al. (2018) [85]	Freshwater	Invertebrates and human-induced contamination	Ecological monitoring	eDNA is not only applied for biodiversity monitoring but can be promising tool for understanding the impact of human-induced contamination in river ecosystems.
Ushio et al. (2018) [87]	Freshwater	Bird communities	Avian biodiversity patterns	eDNA metabarcoding method can serve as an essential alternative for taking a snapshot of bird diversity and potentially can be effective for ecosystem conservation and management.
Ramírez et al. (2018) [83]	Sediments	16S rRNA extracellular genes	Biomonitoring	Extracellular 16S rRNA genes do not greatly influence the overall composition, abundance, and community richness.
Sansom & Sassoubre, (2017) [88]	Freshwater	*Lampsilis siliquoidea*	Quantification	eDNA approach holds tremendous potential for biomonitoring of species and can act as a complementary tool to protect the biodiversity.
Apothéloz-Perret-Gentil et al. (2017) [81]	Freshwater and streams	Epilithic samples	Benthic diatoms index	Taxonomy free molecular index can potentially extend its gauge and frequency to compliment current morphology-based methods for environmental biomonitoring.
Rees et al. (2017) [89]	Freshwater	*Triturus cristatus*	Species-specific identification	Environmental DNA has great proficiency and reproducibility in species-specific detection.
Klymus et al. (2017) [90]	Freshwater	Invasive species and native species	Biodiversity monitoring	The technique of eDNA can enhance identification and conservation efforts of native species and eradicating invasive species.
Deiner et al. (2016) [14]	Freshwater	Metazoan eukaryotes microinvertebrates	Biodiversity patterns	eDNA evaluates the biodiversity and ecological data over an entire landscape.
Guardiola et al. (2016) [33]	Marine	Deep-sea communities	Spatio-temporal biodiversity monitoring	eDNA can be a cornerstone for biomonitoring of deep-sea communities.
Valentini et al. (2016) [72]	Freshwater Marine	Amphibians bony fish	Aquatic biodiversity monitoring	For rare and secretive species, eDNA metabarcoding is the most proficient tool. Such an approach is crucial to address the fundamental and applied research question in ecology.
Thomsen et al. (2016) [22]	Sea water	Fish	Biodiversity monitoring	Application of eDNA for biodiversity assessment can be potentially beneficial not only for marine fish biomonitoring but also for science, society, and the global economy.
Davy et al. (2015) [91]	Freshwater	Sympatric turtles	Biomonitoring of threatened species	eDNA approach could provide a rapid and cost-effective alternative for the detection of freshwater turtles.
Willerslev et al. (2014) [94]	Arctic	Circumpolar plant diversity Nematode diversity	Arctic vegetation history by SedaDNA	eDNA in conjunction with dating methods can reflect information about the vegetation response to glacial climates.
Calvignac-Spencer et al. (2013) [23]	Forest	Mammalian diversity	Species biomonitoring	Caryion fly-derived DNA can be used to address the research questions pertaining to mammalian biodiversity.
Takahara et al. (2013) [92]	Ponds	*Lepomis macrochirus*	Distribution of invasive species	Distribution or presence of invasive species can be estimated more precisely based on eDNA as compared to traditional methods.
Taberlet et al. (2012) [93]	Soil Water	Multi-specific	Biodiversity assessment	Environmental DNA metabarcoding has massive potential to increase data acquisition in biodiversity exploration.
Dejean et al. (2012) [24]	Pond	*American bullfrog*	Species detection	eDNA method is valuable for species detection and surpasses survey methods in terms of sensitivity and sampling effort.
Darling & Mahon (2011) [42]	Freshwater	Invasive Asian carp	Biological invasion	eDNA technique is highly effective for the monitoring of aquatic invasive species.
Chariton et al. (2010) [25]	Estuarine sediments	Eukaryote ribosomal DNA	Ecological assessment	Next-generation pyrosequencing has the ability to identify and enumerate eukaryote species assemblages.
Hebsgaard et al. (2009) [26]	Permafrost	Dirt DNA	Archaeological context	Ancient DNA (aDNA) preserved in sediments can provide insights about the palaeoenvironmental conditions.
Ficetola et al. (2008) [57]	Freshwater	Frog (*Rana catesbeiana*)	Species-specific detection	Development of eDNA contrivance has opened new perspectives for biodiversity monitoring from environmental samples.

### 4.3. Relation between eDNA and Palaeoenvironments

**Figure 4:**
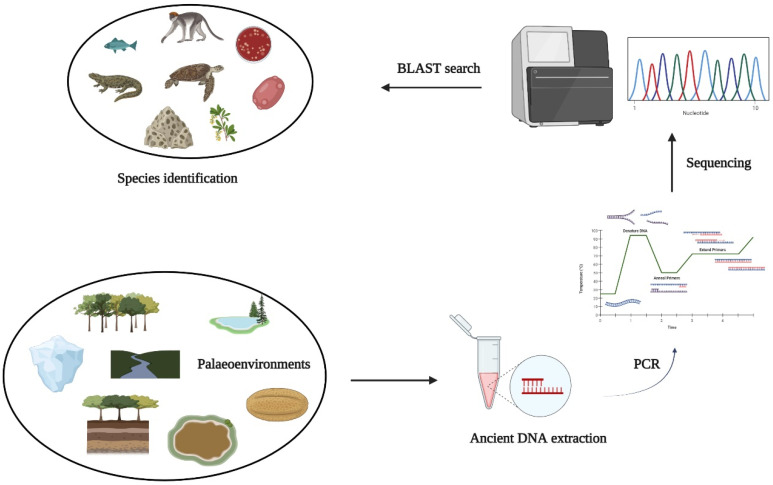
Processes involved in palaeoenvironmental reconstruction and species identification through eDNA.

Palaeoenvironmental DNA is expanding as a dynamic and prominent method in quaternary and archaeological research for reconstructing ancient environments (Figure 4) [95]. Although the first inquiry on palaeoenvironmental DNA was available in 1998 [96], some key matters regarding the diverse origin(s) of DNA and its state affecting eDNA extraction efficacy, stratigraphic consistency, and degradation of DNA for the reconstruction of palaeoenvironments remain a point of concern. The concept of eDNA, in conjunction with dating, is a fascinating method for reconstructing historical environments, be it paleoecology or archaeology [97]. However, to chronicle the palaeoenvironment, applying the notion of eDNA is not as straightforward as it seems.

Utilizing eDNA with dating techniques, marine and lacustrine sediment signatures have an inordinate perspective about past evolutionary events. Different dating techniques can be employed on palaeoenvironmental DNA samples to validate the chronological overview for understanding the response of diverse flora, fauna, and microbes to natural and human influences over time (Table 3 and Table 4). Table 3 indicates the different dating methods, age range, and materials on which these can be carried out. Table 4 gives information on different taxa that can be dated in different ecosystems/habitats.

To reconstruct palaeoenvironments and evaluate ecosystem change through time, bacteria, animals, and plants are utilized in palaeoecological research utilizing palaeoenvironmental DNA: silt-rich ice (dated 450–800 ka) of Central Greenland was investigated by [47], reporting the existence of mixed flora and fauna during a key ice retreat phase before it was subsequently sheltered by ice. Similarly, palaeoenvironmental DNA has been extracted from frozen plant material (dated 4500–5200 cal a BP) in southeastern Peru by [159]. The outcomes revealed ice-free vegetation before the climatic condition in the area altered in the Mid-Holocene. This analysis further indicated that pre-glacial vegetation was distinctive of wetland settings. The investigation carried out by D Costa et al. (2011) isolated and characterized genes from past bacterial DNA from ice-covered sediments aged 30,000 cal (approximately) BP (before present) to confer that antibiotic resistance is a regular occurrence in such ecosystems. Palaeoenvironmental DNA of coprolites and their preserved gut matter has been studied by [52,175,176,177] to reconstruct palaeo diets of extinct fauna. This research further revealed that it is feasible to map parasite abundance geographically.

#### 4.3.1. Lake Sediments

In the coming years, lake sediments will epitomize the utmost utilized material for capturing past events. Capo et al. (2021) and Parducci et al. (2017) have revealed that lake sediments provide an all-inclusive and comprehensive understanding of past diversity over a range of spatio-temporal scales. Similarly, an investigation undertaken by Garcia- Rodriguez et al. (2021) and Giguet-Covex et al. (2015) indicated that sediments obtained from different depths provide favorable settings for DNA-based time series. Over a period of time, lake sediments have been affected by anthropogenic climate change, resulting in prominent changes in species composition, as indicated by the studies of Stein et al. (2020) and Yaccoz et al. (2012). Different studies have been carried out over a period of time to evaluate the efficacy of pollen analysis and ancient sediment DNA (SedaDNA). One such study was carried out by Wilmshurst et al. (2014) in a northern New Zealand offshore island, where sediment cores were retrieved. The investigation confirmed that ancient DNA (aDNA) divulged a better dataset about the indigenous occurrence of certain taxa. Taberlet et al. (2012) and Giguet-Covex et al. (2011) studied ancient plant and mammalian diversity in lake Anterne in northern France using species-specific primers via eDNA. These investigations revealed that the amalgamation of environmental DNA and dating techniques can be used to reconstruct different plant communities over time. Results from such studies have confirmed that lake sediments can dispense eDNA-based time series covering not only plant communities but also domestic animals. Application of environmental DNA on lake sediments followed by pollen analysis using species-specific primers should be utilized to distinguish most all-encompassing signals from the environment [178,179]. As pollen analysis suffers from production and dispersal variables, the investigation by Wood et al. (2008) confirmed that such bias could be augmented by employing proxies such as geographical dispersal statistics and historic vegetation checklists. Very few studies have compared sediment DNA with macrofossils as it is limited by the nature and conditions of the sampling sites, taxonomic group, and the amount of biomass produced [180]. However, in recent years, due to rapid advancement in reference libraries and improved molecular methods, the detection of taxa in ancient sediment DNA has greatly increased with higher taxonomic overlap between DNA and pollen. As per the study conducted by Zinger et al. (2019), the success of recovering the targeted ancient DNA (aDNA) from the sedimentary archives is influenced by the taphonomy and preservation of DNA molecules and DNA extraction, sequencing, and taxonomic identification. Therefore, careful consideration should be kept in mind while performing ancient DNA (SedaDNA) analysis.

#### 4.3.2. Permafrosts and Midden Material

Permafrosts have also been used as representative samples for environmental reconstruction. However, to properly reconstruct the palaeoenvironment in permafrost, it is pertinent to merge many permafrost samples containing a tiny segment of adjacent biodiversity [84]. The first investigation regarding the usage of permafrost samples for evaluating the evolution of plant communities over the course of the past 50,000 years was carried out in the Arctic by [94]. Radiocarbon dating was employed to date around 242 permafrost samples, accompanied by DNA extraction and amplification using plant metabarcodes. For taxonomic recognition of three families, Poaceae, Asteraceae, and Cyperaceae, the universal primer Sper01 pair targeting Spermatophyta and complementary primers Poac01, Aste01, and Cype01 were used. This study revealed that flora was quite diversified prior to the last glacial maximum, revealing 109 plant molecular taxonomic units (MOTUs) and decreasing to about 45 MOTUs. However, with the temperature upsurge during the post-glacial period, 73 MOTUs were observed, with a shift towards graminoids (Poaceae and Cyperaceae) and shrubs. Non-graminoids herbaceous plants (forbs) expectedly dominated about 10,000 years ago for the considered periods. Apart from this, the megafaunal diets of four woolly mammoths, based on their intestinal/stomach contents and coprolites, have also been investigated. It was found that all samples were mostly dominated by forbs. This indicates that forbs were an essential component of their diet. Similarly, Roy-Leveillee (2015) confirmed that under absolute conditions, the theoretical limit of aDNA is ca (approximately) 1 million years in permafrost and ice. Willerslev et al. (2007) reported that for permafrost and ice palaeoenvironmental DNA, the existing practical maximum is up to 400–800 ka. As DNA conservancy is locale-restricted and profoundly affected by the thermal account of material, ref. [181] revealed that specimens from the ice-covered area have the maximum ascendancy rate for palaeoenvironmental DNA isolation.

It has also been found that midden material from archaeological sites epitomizes an imperative cradle of information to assess the food habits of primordial plant communities [182]. It dispenses knowledge about biodiversity by employing humans as biodiversity samplers, as evident from investigations utilizing leeches [183] or carrion flies [23] as an indirect provenance of mammalian DNA [184].

## 5. Uncertainties Associated with eDNA Analysis and Potential Elucidations

The single problem of principal prominence that confronts the approval of eDNA investigations is the ambiguity concerning false positives (detect species when no target species eDNA is present in the sample) and false negatives (fails to detect species when target species DNA is present in the sample). For investigations targeting invasive, rare, or endangered species, false negatives are a notable obstacle while false positives are reflective of sample contaminations, also create ambiguities during the various steps of sequencing assignments [185]. Therefore, to counteract such limitations, this paper has listed a few possible elucidations and solutions to optimize the eDNA technique for wide-spectrum detection of biodiversity. Table 5 shows the different limitations of environmental DNA techniques with their possible solutions and elucidations.

## 6. The Way Forward

For the eDNA technique to truly take off, the present methods employed for ecological assessments would be needed to be adopted to the eDNA metabarcoding framework. As exploration encompassing eDNA investigations is increasing, it can be anticipated that primers for further species will be available. There will be a tremendous build-up of knowledge on eDNA influenced by perseverance and its dispersion in diverse environments, helping to understand DNA quantities and densities in organisms as sampling protocols improve. eDNA can be an essential technique for the surveillance of endangered species. Utilizing three-way approaches viz the traditional approach, taxonomic, and ecological proficiency approach with the dominant potential of eDNA can deliver the results that could be fruitful for efficient and quick species preservation and monitoring. eDNA is vital for the provenance of temporal ecological data. While eDNA is progressively prompted as a method for monitoring, its worth for hypothesis-driven temporal ecological research can prove just as consequential. Similarly, the notion of eDNA can expedite the depiction of the environment–organism interactions. Deterioration of bee species globally and colony collapse are disadvantageous to universal food production. Utilizing eDNA with NGS can deliver a simple, accurate, fast, and robust tactic to ascertain the geographical foundation of plant DNA existing in honey samples. Similarly, spatio-temporal variation in the feeding behavior of species (diet analysis) has not yet been investigated regularly, possibly due to logistic constraints. However, the eDNA approach has been utilized for analyzing the prey content of nearly 2000 fecal samples in France [195]. This investigation reported that much diversity in species diet is contrary to what has been stated in the literature. This is a prime illustration of how eDNA technology can enhance our understanding of elusive species. Beyond diet characterization, eDNA can provide more exhaustive characterization than other methods. The feces and stomach contents can further be explored for investigating resource portioning, planning conservation measures, range and habitat use, ecological impressions of varying populations, and human dimensions.

Research on environmental DNA has seen a serious progression and expansion over the years, and with time, now the scientific community is applying the concept of eRNA alongside eDNA. Researchers during the COVID-19 pandemic have utilized eRNA analysis to track large-scale outbreaks of diseases in wastewater. Such detection of SARS-CoV-2 prior to medical detection of human outbreaks amplified promptly, providing progressive warning of a surge in infected individuals. With such advancing technology, pivotal and limited medical resources can be provisioned in areas of most need. The advantages of eDNA are not limited to the uncovering of human pathogens. In the immediate future, such a technique will benefit in understanding the behavior of pathogens that creel biodiversity conservation efforts (e.g., turtle-specific DNA virus).

Environmental DNA has a wider application in citizen science, where citizens can be engaged in the know-how of commercially available sampling kits. This can help involve people in biodiversity sampling in a way that could complement existing traditional monitoring methods. As such, a more user-friendly method of data analysis is needed. In the near future, it may be possible to automate eDNA sampling, but it needs further research to investigate the temporal longevity and spatial dispersal of eDNA in diverse ecosystems. Therefore, further assessments are needed to evaluate the factors that affect eDNA presence, perseverance, and dispersal in dissimilar environments.

We want to underline that eDNA techniques will supplement conventional monitoring rather than replace it. This is obvious from the sediment literature, where macrofossil, pollen, and traditional flora surveys supplement SedaDNA (sediment ancient DNA), and eDNA has a lower detection probability for certain freshwater species. Furthermore, the last procedure of eDNA studies (which is difficult to standardize) is the interpretation of the data, and the need for well-trained taxonomists and ecologists to effectively interpret results for recommending future actions. The future of environmental DNA technology is bright with its immensely exceeding potential in comparison to traditional biodiversity monitoring methods. With the advancement of technology, the eDNA method may become a commonplace method for biodiversity monitoring, but there is a definite need to let such a technique mature as it is still a developing field.

## 7. Conclusions

The findings of the present review indicate that environmental DNA is quickly emerging as an important apparatus for expeditious research in ecosystem management.

Analysis of the studies carried out in the last decade reveals that compared to the traditional methods, the detection probabilities employing eDNA exertion are considerably higher, predominantly when targeting rare and secretive species.

The present study reveals that the environmental DNA technique has found a wider application in aquatic systems followed by marine and terrestrial ecosystems to fishes followed by invertebrates.

Environmental DNA can be significantly inexpensive and consumes less time. As out taxonomic understanding is becoming more meager, the data reveals that specific primers utilized for species documentation could provide better taxonomic tenacity than conventional methods.

The current study has demonstrated that the environmental DNA procedure has an incredible prospective for approximating population abundance, which is essential, particularly for surveying invasion by harmful, rare, and threatened species.

This review further establishes that careful site selection, sampling design, and reliable stratigraphy become critical for palaeoenvironmental reconstructions as post-depositional reworking and DNA leaching can diminish its efficacy as a palaeoecological contrivance.

Furthermore, this paper summarized and discussed the different dating techniques that can be applied with environmental DNA to provide more insight into past ecosystems. However, issues that ensue during its application need further attention to streamline and authenticate the methodologies to remove ambiguities during the various phases of eDNA application.

## Figures and Tables

**Table 1:** Literature search based on specific keywords utilizing scientific databases.

Search Word	Search Field	Number of Hits in Major Data Bases		Last Updated
		PubMed	Scopus	
“eDNA”	Article, title, keywords	1270	1640	31 July 2021
“eDNA and aquatic”	Article, title, keywords	290	169	31 July 2021
“eDNA and freshwater”	Article, title, keywords	141	157	31 July 2021
“eDNA and sediments”	Article, title, keywords	51	71	31 July 2021
“eDNA and diversity”	Article, title, keywords	125	163	31 July 2021
“eDNA and palaeoenvironmental reconstructions”	Article, title, keywords	39	59	31 July 2021

**Table 3:** An overview of dating techniques that can be used in tandem with environmental DNA.

Method	Range	Materials	References
**Radioisotopic**			
^14^C	35 ka	wood, shell	[98,99,100]
U/Th	10–350 ka	Carbonate (corals, speleothems)	[101,102]
Thermoluminescence (TL)	30–300 ka	quartz silt	[103,104]
Optically Stimulated Luminescence	0–300 ka	quartz silt	[105,106]
**Cosmogenic**			
In situ ^10^Be, ^26^Al	3–4 Ma	Quartz	[107,108]
He, Ne	Unlimited		[109,110]
^36^Cl	0–4 Ma	Olivine, quartz	[111,112]
**Chemical**			
Tephrochronology	0–several Ma	Volcanic ash	[113,114,115]
Amino acid racemization	0–300 ka; range temperature reliant	Carbonate shell	[116,117,118,119]
**Paleomagnetic**			
Identification of reversals	>700 ka	Fine sediments, volcanic flows	[120,121,122]
Secular versions	0–700 ka	Fine sediments	[123,124,125]
**Biological**			
Dendrochronology	10 ka, subject to indigenous master chronology	Wood	[126,127,128,129]

Abbreviations used: ka (thousand years); Ma (million years).

**Table 4:** A sample of research publications that focused on the congruency between eDNA and palaeoecology.

Material	Target Taxa	Age Range	References
Peat	Plantae	155 ka	[130,131,132]
Permafrost	Bacteria, fungi, bryophyta, plantae, insecta, mammalia, aves	2–<600 ka	[133,134,135,136,137,138,139]
Ice	Fungi, protista, plantae, insect	0.3–<800 ka	[47,139,140,141,142]
Lacustrine	Diatoms, plantae, crustacea, copepod	13 cal ka–modern	[143,144,145,146,147,148,149]
Cave deposits	Plantae, insecta, mammalia, aves	10.8–0.6 ^14^C ka	[150,151,152,153]
Marine	Foraminifera, radiolarian, plantae	≤45 ka	[154,155,156,157,158]
Glacial (fluviogravel and moraine)	Plantae	4.5–5.2 cal ka	[159,160,161,162]
Soil	Plantae, mammalia, Aves	5.5 cal ka–modern	[163,164,165,166,167]
Rodent, midden	Plantae, vertebrata	10.1 ^14^C ka	[168,169,170,171]
Coprolites	Plantae, parasites, mammalia, aves	32–06 ^14^C ka	[164,172,173,174]

Abbreviations used: ka (thousand years); cal (approximately).

**Table 5:** Problems associated with eDNA-based species identification and potential possible solutions.

Problem/Limitation	Elucidation of Problem	Methods Affected	Possible Solutions	References
**Wrong method**	Detection possibility of eDNA and estimation of biodiversity is affected by field, laboratory, and bioinformatics protocols, thereby making it obligatory to select an optimal protocol as imperfectly designed methodology impacts the results.	Microarrays, PCR, qPCR, and Metabarcoding	Execution of the relative field findings and the laboratory procedures. Adoption of validated and justified means through exploration of mock communities	[186,187,188,189]
**False positives**	Improper handling of samples, lacking adequate specificity of primers and probes, errors in data analysis, and mutations that accumulate post-mortem produce false positives.	Microarrays PCR qPCR Metabarcoding	Include positive and negative controls for appropriate optimization of protocols, expending numerous markers or primers, choose suitable factors for bioinformatics scrutiny of sequences.	[35,42,190,191]
**False negatives**	False negatives arise owing to prompt degradation or limited eDNA amount in samples. Primer bias can also result in false negatives.	Microarrays PCR qPCR Metabarcoding	To confirm the sampling size, the accumulation curve of species can be generated to attain an asymptote; furthermore, multiple PCRs on each extract can be conducted, appraisal of results in contrast to customary community composition assessments.	[42,187,190,191,192,193]
**Partial ecological evidence**	Owing to deficiency of information about the sex and size of the individuals distinguished by eDNA.	Microarrays PCR qPCR Metabarcoding	Life stage and sex certain markers can be used to overcome limitations of such nature.	[72]
**Limited taxonomic resolution**	Uncategorized diversity and its poor linkage to their ecology.	Barcoding Metabarcoding	When few target species are investigated, utilize local reference libraries. Employ global reference sequence	[35,193,194]

## Data Availability

Not applicable.
